# TNF-R2 expression in acquired middle ear cholesteatoma

**DOI:** 10.1590/S1808-86942011000400020

**Published:** 2015-10-19

**Authors:** Rodrigo Faller Vitale, Celina Siqueira Barbosa Pereira, Adriana Leal Alves, Jose Humberto Tavares Guerreiro Fregnani, Fernando Quintanilha Ribeiro

**Affiliations:** 1 PhD, preceptor; 2 PhD in Medicine, Assistant Professor at the Department of Morphology of the Medical Sciences School of Santa Casa de São Paulo; 3 PhD in Medicine, Professor at the Department of Morphology of the Medical Sciences School of Santa Casa de São Paulo; 4 PhD in Medicine, Professor at the Graduate Program in the Fundação Antônio Prudente - Hospital do Câncer A. C. Camargo; 5 PhD in Medicine, Adjunct Professor - Department of Otorhinolaryngology - School of Medical Sciences - Santa Casa de São Paulo

**Keywords:** bone resorption, cholesteatoma, middle ear, receptors, tumor necrosis factor, type II, tumor necrosis factor-alpha

## Abstract

**Abstract:**

Acquired middle ear cholesteatoma is a disease which promotes bone erosion resulting in potentially serious complications. The tumor necrosis factor alpha (TNF-α) is present in cholesteatoma and it is related to bone erosion, as shown by different authors. To understand the aggressiveness characteristics of cholesteatoma is necessary, however, to better address the presence and distribution of their receptors.

**Objective:**

To evaluate the expression of type 2 TNF-α receptor (TNF-R2) in fragments of cholesteatoma and correlate it to the degree of inflammation present.

**Material and methods:**

observational cross-sectional study, which analyzed 33 fragments of cholesteatomas through histological analysis and immunohistochemistry (using as primary antibody to TNF-R2 LabVision ® brand). The evaluation was performed by means of a qualitative and semi-quantitative agreement with the observed intensity. For statistical analysis we used the Fisher exact test and Spearman's correlation coefficient (considered statistically significant when *p* ≤ 0.05).

**Results:**

The expression of TNF-R2 was present in all fragments, however a statistical analysis showed no correlation or association between inflammation and the expression of TNF-R2.

**Conclusions:**

TNF-R2 is present in cholesteatoma of the middle ear, however, its expression is not directly related to the degree of inflammation observed in patients with this disease.

## INTRODUCTION

The middle ear acquired cholesteatoma was first described by, Curveilhier, in 182^9^,^1^, and it is characterized by tympanic cavity invasion by a keratinized squamous epithelium, which is different from the columnar pseudostratified ciliated epithelium, with goblet cells present near the auditory tube or the simple, cubic or columnar squamous cell epithelium - of the remaining middle ear^2^. Histologically, cholesteatomas may be broken down into matrix (epithelium) and peri-matrix (underlying connective tissue)^3^.

The cholesteatoma matrix has four distinct layers: basal, spinous, granulous and strata cornea, as well as the thin skin. The peri-matrix is characterized by the presence of a loose connective tissue made up of collagen and elastic fibers, fibroblasts and inflammatory cells^4^. Histological analysis of the cholesteatoma matrix may show different patterns: atrophy, acanthosis, basal layer hyperplasia and epithelial cones. It is common to find many of these patterns occurring at the same time in the same patient^2^.

Because of its capacity to cause bone erosion, present in 80% of the cases^5^, the cholesteatoma is responsible for extracranial and intracranial complications. These complications, when present, carry a high rate of morbidity and mortality^6^. Hence the relevance of studying the mechanisms through which acquired middle ear cholesteatomas cause bone erosion.

The early 90's saw the publication of the first papers suggesting that TNF-α could be an important cytokine involved in the bone destruction process^7,^^8^. The cytokines released in the inflammatory process present in the peri-matrix would be the ones responsible for the bone destruction seen in cholesteatomas^9^. Among these, TNF-α stands out as one of the main cytokines involved in this process, working together with the Receptor Activator Nuclear Kappa B Ligant (RANKL) and interleukins 1 and 6, to cause bone destruction and remodeling^10^. Therefore, we stress the importance of studying the expression of TNF-R2 and its association with the inflammatory process.

TNF-R2 has been described in different organs, e.g. the kidney, brain and ovary, being always associated with the inflammatory process because of the activation of the Kappa B nuclear factor (NF-kB)^11^, ^12^, ^13^. Notwithstanding, there is still no description of TNF-R in cholesteatomas.

The two TNF-α receptors have similar activities. They are involved in the inflammatory process, being associated with bone erosion and they can trigger apoptosis (programmed cell death), the latter is especially associated with the TNF-R2. However, the mechanism responsible for establishing which effect will prevail is still not totally clear^13^.

## OBJECTIVES

The goal of the present study was to assess the expression of the type-2 tumor necrosis factor alpha receptor (TNF-R2), by means of using immunohistochemistry techniques in cholesteatoma fragments acquired from the middle ear and correlate them with the degree of inflammation present.

## MATERIALS AND METHODS

This is a cross-sectional, observational study, approved by the Ethics Committee of the Medical School of Santa Casa de Misericórdia de São Paulo, where it was carried out. The material encompassed 68 cholesteatoma fragments taken from 68 patients submitted to ear surgery in order to remove the acquired cholesteatoma from the middle ear. The surgeries were done in the Department of Otorhinolaryngology of this Institution from August 2007 through March 2009. The disease diagnosis was based on the clinical history and physical exam of the patients. The surgical procedures were done by different otorhinolaryngologists.

From this study we extracted those fragments which did not have an epithelium (matrix), visible upon light microscopy, dyed by hematoxylin-eosin (HE), and 35 fragments were excluded. This way, the total number of fragments used in this study was 33.

After removing the cholesteatoma fragments, the material was fixed in 10% formaldehyde and processed by conventional histology techniques, with paraffin inclusion. For histology purposes, we considered the following patterns: atrophy (matrix with a thickness of a maximum of four layers of keratinocytes), acanthosis (matrix thickness increase in lieu of the spinous layer), basal layer hyperplasia (increase in the number of basal keratinocyte layers), epithelial cone formation (matrix invaginations to inside the peri-matrix) and inflammatory process present in the peri-matrix. The assessment was carried out in qualitative (present or absent) and semi-quantitative fashions, grade varying between 0 and 3 according to the intensity observed (absent = 0; weak = 1; moderate = 2 and severe = 3).

For immunohistochemistry we used the primary TNF-R2 polyclonal primary antibody synthetized from rats (Labvision^U+000AE^, USA), in the title of 1:100. The secondary antibody used was the Max Polymer Detection System (New Kit Link, Novocastra®, UK).

**Figure 1:**
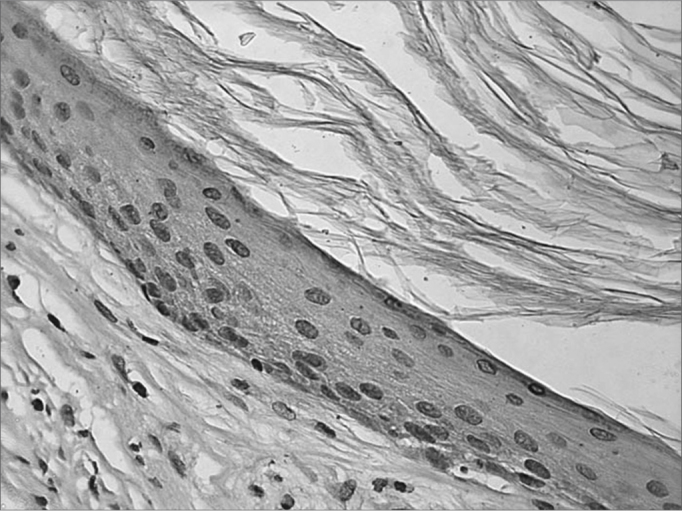
Microphotography from a cholesteatoma fragment, in which we see a weak reaction against the TNF-R2 receptor in the cytoplasm of the matrix keratinocytes (IHQ X400).

**Figure 2:**
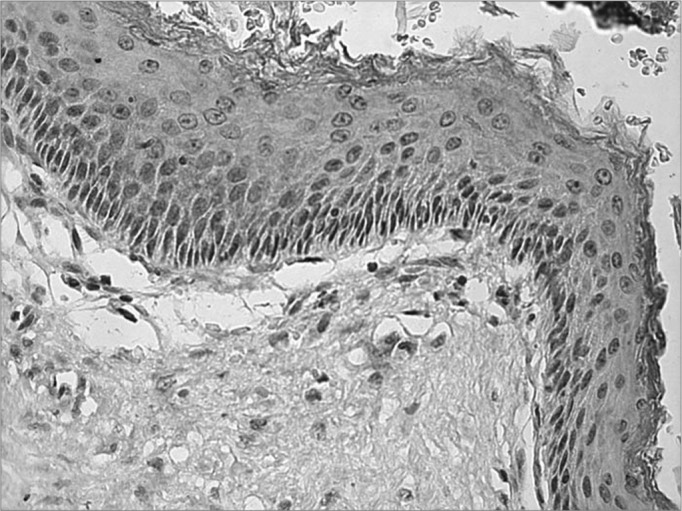
Microphotography of the cholesteatoma fragment, in which we notice moderate reaction against the TNF-R2 receptor in the cytoplasm of the matrix keratinocytes (IHQ X400).

**Figure 3:**
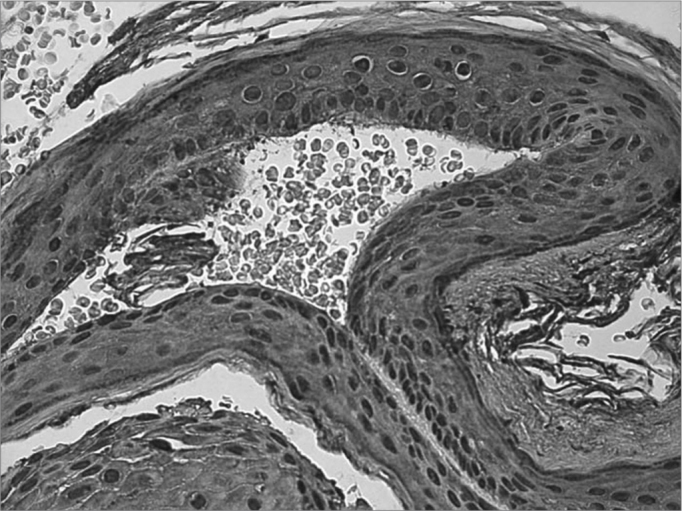
Microphotography of the cholesteatoma fragment where we see a severe reaction against the TNF-R2 antibody in the cytoplasm of matrix keratinocytes (IHQ X400).

The immunohistochemical evaluation was carried out in a quantitative form (present or absent) and semi-quantitative, graded between 0 and 3 according to the intensity of the color observed: absent = 0; weak = 1(Figure 1); moderate = 2 (Figure 2) and severe = 3 (Figure 3).

The slides with cholesteatoma fragments were analyzed under a light microscope model Axioscope 40 (Carl Zeiss - Brazil), with a 10X eye piece and 10X, 20X and 40X magnification lens for histological evaluation and also for immunohistochemistry. All the slides were assessed by three independent observers, in a blinded way. The microscope was coupled to an Axiocam MRc 5 (Zeiss) camera in order to obtain the digital images of the material, using the Axiovision 4.8 software.

After data collection we setup a computerized database which was submitted to statistical analysis, with the help of the Statistical Package for the Social Sciences (SPSS) version 13.0 software.

We carried out a descriptive analysis, calculating minimum and maximum values, mean, median and standard deviation for the age variable and calculations of the absolute and relative frequencies for gender and for the histopathological and immunohistochemical variables.

To study the association among the qualitative variables we used the Fisher's exact test. In order to check for the correlation among the quantitative variables we used the Spearman Coefficient.

All the tests were carried out in a bicaudal way and the significance level adopted was 5%.

## RESULTS

We studied 33 cholesteatoma fragments from 33 patients submitted to ear surgery, 17 of them (51.5%) were men and 16 (48.5%) women. Minimum age was seven years, and the eldest patient was 70 years old, with mean age of 30 years, median of 31 years and standard deviation of 16.4. Of the 33 patients included in the study, two (6.1%) did not know how long it was between symptom onset and admission at the Department of Otorhinolaryngology for surgery. Of the 31 patients who reported it, 13 said they had had the disease for less than five years (41.9%), seven reported between six and ten years (22.6%), nine mentioned a time span between 11 and 20 years (29%) and, only two patients reported having had the disease for over 20 years (6.5%). Time between symptom onset and admission to surgery at the Department of otorhinolaryngology varied between one and 60 years, with a mean of 11.5, median of eight years and standard deviation of 11.6 years.

In regards of the ear affected by the disease, four patients (12.1%) had chronic cholesteatomatous otitis media in both ears, and 29 (87.9%) had unilateral involvement; 15 (45.5%) in the right ear and 14 (42.4%) in their left ears.

As far as clinical symptoms reported by the patients upon admission goes, 31 (93.9%) reported otorrhea, 30 (90.9%) reported hypoacusis and 27 (81.8%), reported dizziness spells.

Ossicular chain involvement was reported in 28 patients (84.8%), who had erosion in one of the ossicles and, in five (15.2%) the ossicular chain was not seen involved during intraoperative assessment. Considering the ossicle involved, 23 patients (69.7%) had malleus erosion, 23 (69.7%) had incus erosion, and 18 (54.5%) had stapes erosion.

Besides ossicular chain involvement, we noticed the presence of erosion in the semicircular canal, facial nerve exposure and also dura mater exposure. There was semicircular canal erosion in three patients (9.1%), facial nerve exposure in six (18.2%) and dura mater exposure in five (15.2%).

Histological assessment found more than one histopathological pattern (atrophy, acanthosis, basal layer hyperplasia and epithelial cones) simultaneously, in most of the cholesteatoma fragments analyzed.

**Figure 4:**
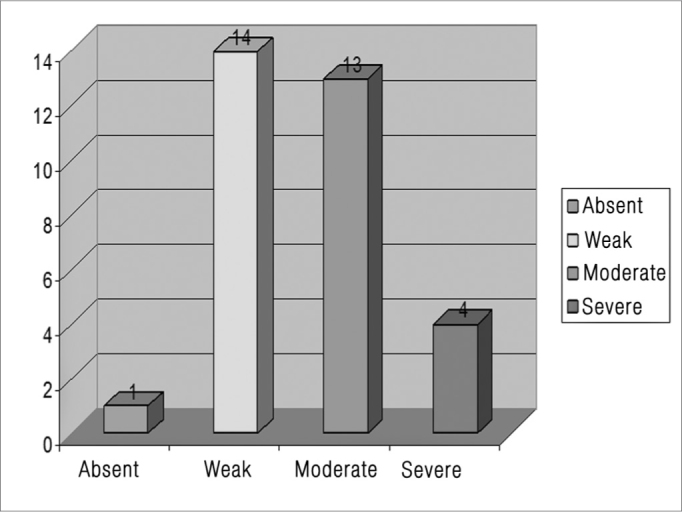
Inflammatory process severity analysis.

Because of the lack of a peri-matrix in one of the 33 fragments investigated, it was not possible to assess the intensity of the inflammation in that case. Of the 32 remaining cases, inflammation was absent in one (3.1%), weak in 14 (43.8%), moderate in 13 (40.6%) and severe in four (12.5%) (Figure 4). The inflammatory infiltrate was mostly lymphoplasmacytic with areas of neovascularization.

**Figure 5:**
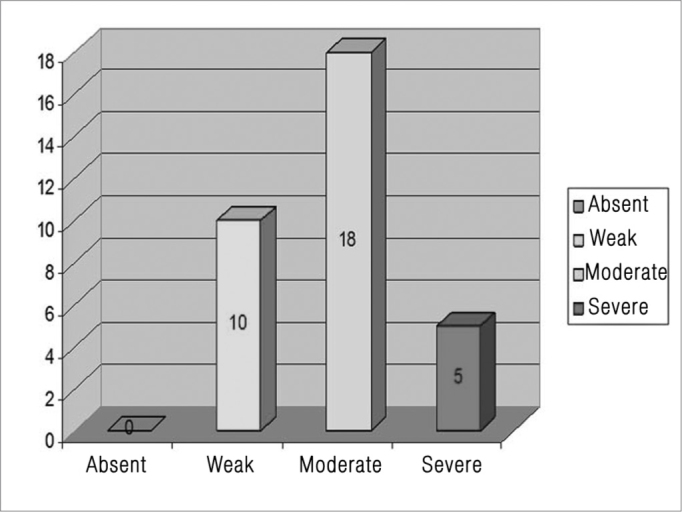
ITNR-R2 distribution in the cholesteatoma fragments analyzed.

Upon immunohistochemical evaluation we observed a reaction from the anti-TNF-R2 antibody in the matrix cells in all the cholesteatoma fragments analyzed. This reaction was considered weak in 10 cases (30.3%), moderate in 18 (54.5%) and severe in five (15.2%) (Figure 5). Besides the cytoplasmic reaction, we also observed reactions in almost all the keratinocyte nuclei from the matrix of the analyzed fragments, and also in the nuclei of inflammatory cells present in the peri-matrix.

The statistical analysis did not show statistically significant association between the degree of inflammation present in the middle ear acquired cholesteatoma and the presence of TNF-R2 (*p*= 0.589). We also did not see positive and statistically significant correlation using the Spearman's correlation coefficient (*p*= 0.155).

## DISCUSSION

The middle ear acquired cholesteatoma is a disease characterized by tympanic cavity invasion by an epithelium (matrix) which is different from the one usually found in the middle ear. Histologically, this epithelium is similar no normal skin^3^.

Histological analysis also showed that the matrix may present with different histological patterns, such as atrophy, acanthosis, basal layer hyperplasia and epithelial cones. These patterns frequently coexist in the same fragment, as shown in the present study. These histological findings are in agreement with what has been published in the literature^2,^^4^.

The evaluation of the peri-matrix from the cholesteatoma fragments reported inflammatory process present in 96.9% of the cases, in different severities. This fact is in agreement with reports from Pereira^4^ (2001) and Alves et al.^2^ (2008), who noticed an inflammatory infiltrate in the peri-matrix in 90.9% and 96.0%, respectively. These results match the clinical history of middle ear acquired cholesteatomas, because it is a disease which, most of the times, happens together with an intense infectious inflammatory process. This inflammatory and infectious reaction is associated with bone erosion and the complications caused by this disease^14^. Nonetheless, the statistical analysis did not show any correlation or association between TNF-R2 expression and the inflammatory process severity.

Cholesteatomas usually cause ossicular chain erosion. In the present study, during surgery, we noticed that the ossicular chain was eroded 84.4% of the patients. These data corroborate literature data, which report ossicular chain erosion on 91.5% of the patients^14^.

In the present study, the most affected ossicle was the incus, especially its long apophysis, and the malleus, both in 23 patients (69.7%). Stapes erosion happened in 18 patients (54.5%). These findings differ from the ones from Tos^15^ and Dornelles et al.^16^ who reported incus erosion as the most frequent, followed by the stapes and, later, the malleus.

The middle ear acquired cholesteatoma is a disease which courses with an intensive infectious and inflammatory process. Such condition induces the release of different substances (cytokines and growth factors) which act together in synergism, resulting in the aggressive cholesteatoma characteristics^9^. Among these substances we stress the interleukins 1, 6 and 8, TGF-α, TGF-β, EGF, KGF and, especially, the TNF-α, considered one of the main cytokines involved in inflammatory and immune processes^17^.

There are two receptors for TNF-α, the TNF-R1 and the TNF-R2. The role of each one of the TNF-α receptors is still unclear^13^.

The goal of the present study was to investigate TNF-R2 expression by means of immunohistochemical techniques in middle ear acquired cholesteatoma fragments and correlate them to the degree of inflammation seen upon histology.

In this study we assessed TNF-R2 expression by means of immunohistochemical techniques in middle ear acquired cholesteatoma fragments. TNF-R2 reaction was cytoplasmic and happened in all the keratinocytes of the matrix from analyzed fragments. There was also reaction in some peri-matrix inflammatory cells (lymphocytes and plasmocytes). Besides TNF-R2 expression, inflammation was also present.

We carried out a statistical analysis in order to check whether or not there was any association or correlation between TNF-R2 expression and the degree of inflammation. However, in our study we did not see TNF-R2 involvement in the cholesteatoma's inflammation. In other words, the TNF-α receptor is present in the middle ear acquired cholesteatoma; nonetheless, its expression (number of receptors) is not associated with the degree of inflammation seen. Notwithstanding, we still need further studies, with larger cholesteatoma samples, in order to obtain a more reliable statistical analysis.

In the present study, besides the cytoplasmic reaction (pattern) arising from the connection between the TNF-α and the TNF-R2, we also detected a significant nuclear expression in all the slides assessed, in epithelial cells of the matrix and in some cells of the peri-matrix inflammatory infiltrate (lymphocytes and plasmocytes). This reaction may indicate a crossed reaction with some cell nucleus proteins; however, this still needs further clarification in future studies.

## CONCLUSIONS

1) TNF-R2 is present in acquired middle ear cholesteatoma matrixes.

2) It was not possible to correlate TNF-R2 expression with the degree of inflammation present in the acquired middle ear cholesteatoma.
